# Polystyrene-Sepiolite Clay Nanocomposites with Enhanced Mechanical and Thermal Properties

**DOI:** 10.3390/polym14173576

**Published:** 2022-08-30

**Authors:** Shafi Ur Rehman, Sana Javaid, Muhammad Shahid, Iftikhar Hussain Gul, Badar Rashid, Caroline R. Szczepanski, Muhammad Naveed, Sabrina J. Curley

**Affiliations:** 1 School of Chemical and Materials Engineering (SCME), National University of Sciences and Technology (NUST), Islamabad 44000, Pakistan; 2 School of Natural Sciences (SNS), National University of Science and Technology (NUST), Islamabad 44000, Pakistan; 3 Department of Chemistry, University of Wah, Quid Avenue, Wah Cantt, Rawalpindi 47040, Pakistan; 4 Research and Development (R & D) Section, National University of Technology NUTECH, Islamabad 44000, Pakistan; 5 Department of Chemical Engineering & Materials Science, Michigan State University (MSU), East Lansing, MI 48824, USA; 6 School of Packaging, Michigan State University, 448 Wilson Rd, East Lansing, MI 48824, USA

**Keywords:** polymeric nanocomposites, polystyrene, sepiolite, mechanical properties, flame retardancy

## Abstract

Polystyrene (PS)/sepiolite clay nanocomposites were prepared via the melt extrusion technique using vinyl tri-ethoxy silane (VTES) as the compatibilizer and cross-linking agent. Mechanical, thermal, and flame-retardant properties of the newly developed polystyrene-based nanocomposites were determined. Surface morphology was investigated using scanning electron microscopy (SEM), examining the distribution of the filler in various compositions of fabricated composites. Structural analysis of the samples was carried out using the Fourier transform infrared spectroscopy (FTIR) and X-ray diffraction (XRD) techniques. Thermal stability was determined by thermal gravimetric analysis (TGA), showing a maximum 30.2 wt.% increase in residue by adding sepiolite clay. The results obtained from the dynamic mechanical analyzer (DMA) in terms of the storage modulus, loss modulus and damping factor exhibited better stress transfer rate and effective interfacial adhesion between the filler and the matrix. The higher filler loaded sample showed greater flame retardancy by decreasing the burning rate up to 48%.

## 1. Introduction

Polymer nanocomposites have captured the interest of many researchers owing to their high performance, remarkable mechanical and thermal properties [1,2,3,4]. They possess outstanding properties, such as improved mechanical strength and dimensional stability, better optical, magnetic, and electrical properties, enhanced water and oxygen barrier, thermal stability, meaningful flame retardancy, chemical resistance, increased anti-scratch and wear resistance properties, etc. [5,6,7,8]. It has also been proven that the properties of the polymer nanocomposites can be improved by employing high aspect ratio and large surface area of the nanofillers. The exploration of such high-performance polymeric materials with superior properties has now become the prime area of interest. Conventional composite materials are now days from being replaced by high-performance nanocomposites due to the lower amount of filler consumption (3–5 wt.%) and other desirable synergistic effects. For the last two decades, researchers have inclined toward using assorted nanofillers, such as graphene, carbon nano tubes (CNTs), nanocellulose, metallic nanoparticles, nano clays, etc. [9,10,11,12,13]. Nano clays are being widely explored, since they are ubiquitous, low cost and impart improved mechanical properties to the nanocomposites. Initially, montmorillonite clay was used in nylon-based composites by Usuki et al. in 1993, capturing the attention of many researchers and motivating them to explore many other formulations [14]. Recently, the trend has been shifted toward needle-like, low-cost sepiolite clay [15]. A considerable amount of published data is available regarding polymer/clay nanocomposites, which address the lamellar layered silicates targeting the montmorillonite and examining the intercalation and exfoliation of the clay; however, polymer/sepiolite nanocomposites is a relatively new area of interest to the researchers.

Polystyrene is broadly used worldwide as a commercial polymer. At present, nanocomposites of polystyrene are prepared by various methods, such as in situ polymerization, solution casting and melt intercalation [16,17,18,19]. These three methods are used discretely or in combination. However, polystyrene is not generally preferred as an engineering plastic because of its higher brittleness [20]. To counter this bottleneck, high-impact polystyrene (HIPS) is used for a wider range of applications in several industries. HIPS contains only 5–8% of butadiene, which helps in increasing the impact resistance and provides greater dimensional stability and excellent machinability [21,22]. These properties facilitate outstanding processing and molding of the materials. However, higher flammability and lower chemical stability in organic solvents are concerning issues, which need to be addressed using specific fillers.

Nano clays are extensively probed for the manufacturing of polymeric nanocomposites. These materials are preferred because of the abundant availability, low cost, and eco-friendly nature [23,24,25]. The clays commonly used with polystyrene are montmorillonite, bentonite, hectorite and kaolinite. The cation exchange capacity of montmorillonite clay usually ranges around 110 meq/100 g; hectorite clay has 120 meq/100 g, and kaolinite clay has 86.6 meq/100 g [26,27]. These clays can be dissolved only in hydrophilic polymers, such as poly (ethylene oxide) and poly (vinyl alcohol). It is essential to convert these clays into organophilic nature to make them compatible with hydrophobic polymers [28,29,30]. For the preparation of nanocomposites, clay should be selected based on its ordinary surface charge. Sepiolite is composed of magnesium silicate Mg_4_Si_6_O_15_(OH)_2_.6H_2_O. It is available in fibers, particles, and fine powder form [31]. It is highly porous in nature with low specific gravity that varies from 0.988 to 1.279 and surface area (95 to 400 m^2^/g) [32].

**Figure 1:**
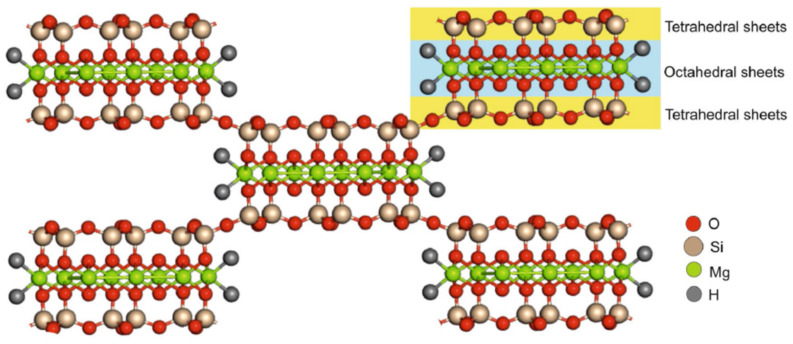
Structure of sepiolite clay. Reprinted/adapted with permission from Ref. [34].

Octahedral magnesium is sandwiched between two tetrahedral sheets of silica. The structural discontinuity of the sheets arises because of the inversion of the Si–O–Si bond, which leads to the formation of tunnels or pores extending along the axis of the fibers [33]. The structure of sepiolite is shown in Figure 1. The silanol group (Si–OH) present at the edges or corners has affinity for the inorganic ions and polymer matrix composites.

## 2. Experimental Section

### 2.1. Materials

General-purpose polystyrene (GPPS-550P) was obtained from Pak Petrochemical Industries Pvt. Ltd., Karachi, Pakistan. Analytical grades of vinyl tri-ethoxy silane (VTES), di-methacrylate, stearic acid, isopropanol, sepiolite clay (SPC) of grade #BCCD4817 and HCl were purchased from Sigma Aldrich^®^ (St. Louis, MO, USA). The antioxidant Irganox 1010 was acquired from Ciba Specialty Chemicals, Basel, Switzerland.

### 2.2. Modification of Sepiolite

The organic modification of sepiolite clay is the first step of prime importance. The successful modification of sepiolite clay helps in reducing the surface energies of silicate layers and plays an important role in increasing the miscibility of silicates in the polymer matrix [35,36]. The modification process starts with the washing of raw sepiolite clay. An amount of 5 g of sepiolite clay was washed in 1000 mL of de-ionized water, adding 2–3 drops of HCl and stirring for 48 h. After stirring, the solution was filtered out using Whatman filter papers (Grade 40), and the residues were vacuum dried at 60 °C overnight. After washing, the modification of the clay was carried out by dissolving 2.5 g of the washed sepiolite clay in 500 mL isopropanol using 1L round bottom flask. The solution was then stirred for 24 h, along with drop-wise addition of 6 mL of vinyl tri-ethoxy silane (VTES) and 2 mL of HCl into it. The solution was then filtered out, washed thrice with methanol and vacuum dried overnight at 60 °C. The obtained modified sepiolite (m-SPC) was preserved in a vacuum desiccator for further use as a filler. Sample codes and details of composition is presented in Table 1.

### 2.3. Composite Preparation

General-purpose polystyrene (GPPS-550P), modified sepiolite clay (m-SPC), antioxidant (Irganox 1010) and stearic acid were poured into a twin-screw extruder (Thermo Haake Poly-lab Rheomix-600, Internal Mixer, Karlsruhe, Germany). Initially, the twin-screw rollers were kept at 60 °C and 10 rpm rotation speed to digest the ingredients. After digestion, the temperature was increased to 210 °C and the rotation speed raised to an optimum speed of 60 rpm. Di-methacrylate (DMC) was added drop wise as a cross-linking agent. Detail of formulation regarding the nanocomposites are shown in Table 2.

### 2.4. Characterization and Analysis

#### 2.4.1. Fourier Transform Infrared Spectroscopy (FTIR)

The structural analysis of the samples was performed by a Fourier Transform Infrared Spectrophotometer (Modal Nicolet 6700 (Thermo Scientific, Waltham, MA, USA). The respective KBr pellets of the samples were produced, and the spectra were obtained by scanning in the range of 4000 to 500 cm^−1^ versus percent transmittance. An average of 16 scans were reported.

#### 2.4.2. X-ray Diffraction Analysis

The X-ray diffraction analysis of the samples was carried out with an X-ray diffractometer (Model: X’ TRA48 Thermo ARL, Tokyo, Japan) using Cu Kα radiation (λ = 0.15406 nm) operating at 45 kV and 40 mA. Radial scans were performed in the reflection scanning mode with 2θ values ranging from 5° to 80° at a scanning rate of 1°/min. The % crystallinity of the samples under investigation was computationally calculated using the XRD deconvolution method, with the following Equation (1).

(1) $$\%\;Crystallinity=\frac{Ic}{Ic+Ia}\times 100$$

where *Ic* and *Ia* are the integrated intensities for the crystalline and amorphous phases in the polymer matrix, respectively.

#### 2.4.3. Morphological Analysis

The surface morphologies of the samples were investigated using a Scanning Electron Microscope (SEM) Model: JSM 6490LA (JEOL, Tokyo, Japan) at 20 kV. The freshly fabricated composite samples were first dried in a vacuum and then gold coated for the surface analysis.

#### 2.4.4. Mechanical Testing 

The tensile properties were determined according to ASTM D-638 using Minebea (model TGI 5kN, Novi, MI, USA). The tensile strength (TS), Young’s modulus (E) and elongation at break (Eb%) of the fabricated samples were measured. The specimens were cut into a dumbbell shape (Dimension: Type 4, Standard: ISO 37:1994) from a 1 mm thick compression molded sheet. Five specimens from each sample were tested, and data were analyzed as mean standard deviation.

#### 2.4.5. Dynamic Mechanical Analysis (DMA)

Pristine polystyrene samples with different loading contents of m-sepiolite were studied using DMA, Discovery Model DMA 850 New Castle, DE, USA. It was used to determine the storage modulus (E′), loss modulus (E″) and damping factor (tan δ). Three-zone bending moment was conducted at a constant frequency of 1 Hz, amplitude of 15.0 μm and temperature range of 25 to 150 °C at a heating rate of 10 °C/min.

#### 2.4.6. Thermal Gravimetric Analysis (TGA)

The thermal stability of the samples was measured using a thermogravimetric analyzer (Model: TGA/SDTA851e, Schwerzenbaclz, Switzerland). A 5 mg sample in an inert environment (N_2_ = 99.999% purity, Grade 5) was placed in an alumina crucible, where the temperature was increased from 20 to 600 °C at 10 °C/min ramp.

#### 2.4.7. Flame Retardancy

For the flame retardancy test, ASTM D4986-20 was followed. The rectangular samples had a length (L) of 150 mm and a width (W) of 50 mm. An average of three samples are reported here. The samples were subjected to the prescribed blue flame of the laboratory burner, according to specification D5025, for 60 s. The burning rates of the samples were determined accordingly.

## 3. Results and Discussion

### 3.1. Structural Analysis

**Figure 2:**
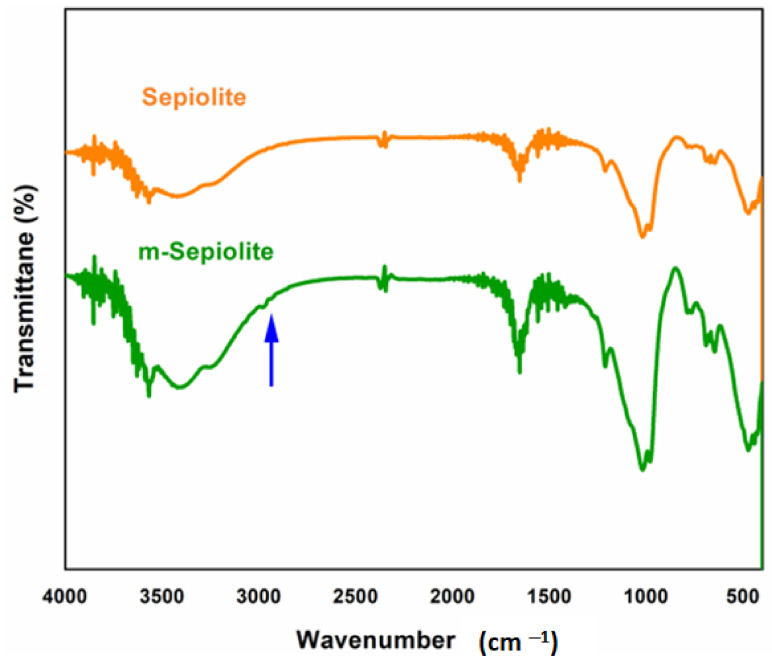
FTIR spectra of pristine sepiolite and modified sepiolite (m-sepiolite) clay.

**Figure 3:**
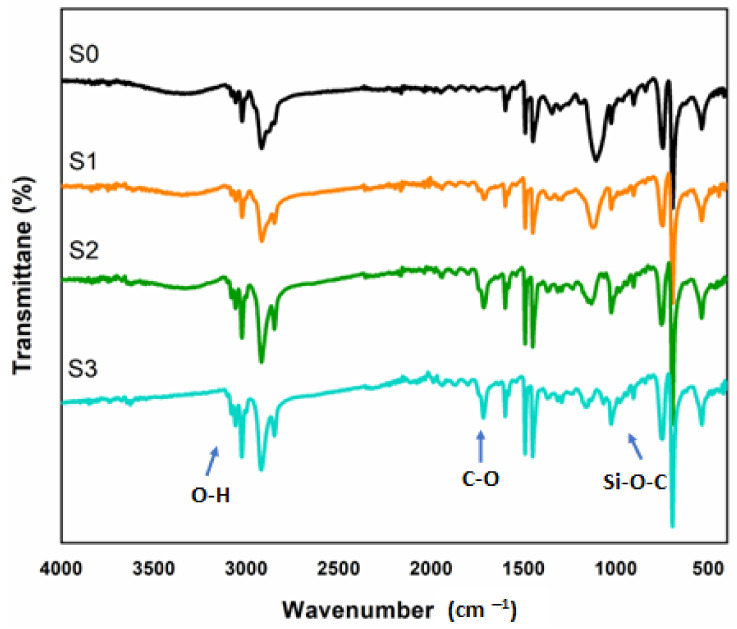
FTIR spectra of S0 (pristine PS), S1 (PS/1 wt.% m- sepiolite), S2 (PS/2 wt.% m-sepiolite) and S4 (PS/4 wt.% m-sepiolite).

FTIR analyses of pristine sepiolite and modified sepiolite (m-SP) are shown in Figure 2, while the spectra of polystyrene/sepiolite nanocomposites are exhibited in Figure 3, respectively. The structural analysis of pristine sepiolite shows the characteristic peak of (H–O–H) stretching vibrations of water molecule appearing in the range of 3650 to 3070 cm^−1^, respectively [37]. The stretching vibrations of (Si–O) are visible at 1210, 1020 and 970 cm^−1^, while (Si–O–Mg) shows bending vibrations at 552 cm^−1^, which can be attributed to octahedral-tetrahedral linkage [38]. Further, the characteristic peaks of sepiolite clay also appear at 1650, 1210, 1020 and 690 cm^−1^. The modified sepiolite (m-SP) spectra show the broadening of O–H stretching vibrations in the region of 3510 to 3650 cm^−1^ and a bending vibration of O–H at 1670 cm^−1^, which can be associated with the successful modification of the sepiolite surface [39]. The Si–O vibrations were also observed at 1210, 954, 771, 695 and 670 cm^−1^ in both pristine and modified sepiolite spectra [40,41]. The siloxane linkage (Si–O–Si) stretching vibrations are visible at 1015 and 471 cm^−1^. The characteristic peak of Si–O–Mg can also be observed at 525 cm^−1^ [42]. As the modification was performed with VTES, a small peak at 2971 cm^−1^ is present, confirming the asymmetrical stretching vibration of the vinyl C–H group.

The FTIR spectra of pristine polystyrene and polystyrene–sepiolite nanocomposites with varying compositions, are shown in Figure 3. Pristine PS (S0) shows the characteristic peaks of C–H stretching vibrations at 3050 and 2810 cm^−1^ for aliphatic and aromatic carbon, while three absorption bands of aromatic C=C stretching vibrations also appear at 1610, 1580 and 1450 cm^−1^ [43]. The corresponding bending vibrations in the fingerprint region of 770–789 cm^−1^ confirm the substitution on the benzene ring due to C–H being out of plane [44].

In Figure 3, PS/m-sepiolite nanocomposites show the absorption bands in the range of 3000–3300 cm^−1^, attributed to the O–H stretching vibration [45]. The characteristic peaks of carbonyl C–O in the range of 1600 to 1712 cm^−1^ are sharper and more prominent in the spectra of S2 and S4 with higher filler contents in the nanocomposites. A sharp band at 1075 cm^−1^ due to Si–O–C appearing in polystyrene nanocomposites confirms the interaction between the Si–OH group of sepiolite and polystyrene [46]. All the related peaks of polystyrene and sepiolite are present, as in the pristine samples.

### 3.2. X-ray Diffraction Analysis

**Figure 4:**
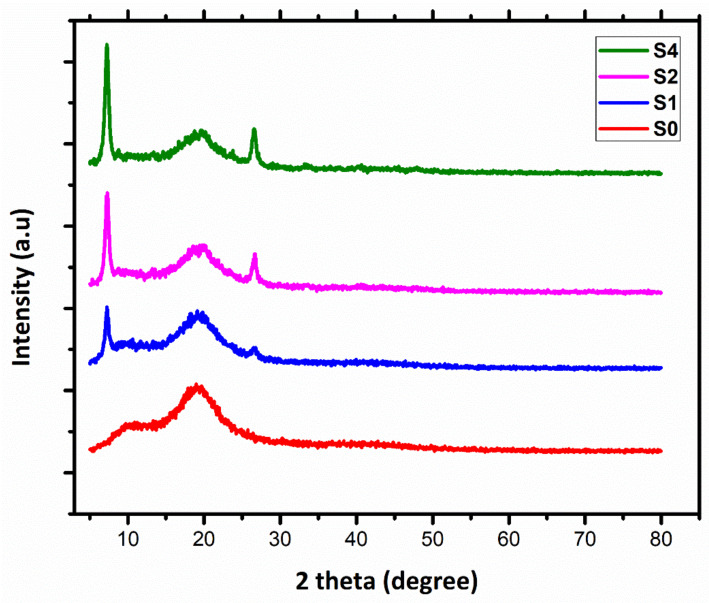
XRD patterns of pristine PS (S0) and PS/m-sepiolite nanocomposites S1, S2 and S4 formulations.

The X-ray diffraction pattern of pristine polystyrene S0 and the effect of adding m-sepiolite as a filler on % crystallinity of polystyrene-sepiolite nanocomposite S1, S2 and S4 formulations are presented in Figure 4. A broad diffraction peak at 20.0° with d = 0.22 observed in S0 shows the amorphous nature of polystyrene [47,48]. The percent crystallinity obtained computationally using the XRD deconvolution method in Eq.1 shows an increase from 35.9% in the S1 sample to 47.3% in the S4 sample because of m-sepiolite clay being added into the polystyrene matrix. This also results in a decrease in broadness of 2θ (X-axis) values at 7.25°, 20.0° and 27.5°, as shown in the XRD pattern. The intense peak at 7.27° with interatomic distance d = 1.22 nm with (110) shows the covalent interaction and exfoliation of clay nanoparticles in the polystyrene matrix and becomes sharper with the increase in the concentration (wt.%) of m-sepiolite. Therefore, the addition of a filler causes an increase in the intra-atomic distance or d-spacing due to the expansion in the polymer matrix, which is attributed to more favorable interactions between the polymer matrix and the filler [49]. The mechanical properties are also related to the level of saturation and dispersion of clay nanoparticles in the polymer matrix. Further, a sharp peak observed at 27.5° (d-spacing = 0.16 nm) evidences the increase in percent crystallinity upon increasing the filler content of m-sepiolite in the polystyrene matrix. These results enhanced the mechanical and thermal properties of nanocomposites, as is also evident in the tensile testing, TGA and DSC results.

### 3.3. Surface Morphology

**Figure 5:**
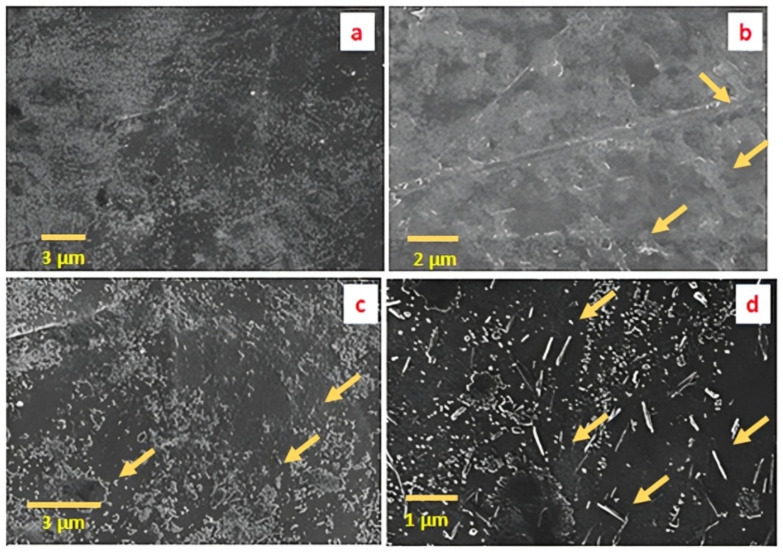
SEM images of S0 (pristine PS) in (**a**), S1 (PS/1 wt.% sepiolite) in (**b**), S2 (PS/2 wt.% sepiolite) in (**c**) and S4 (PS/4 wt.% sepiolite) in (**d**).

**Figure 6:**
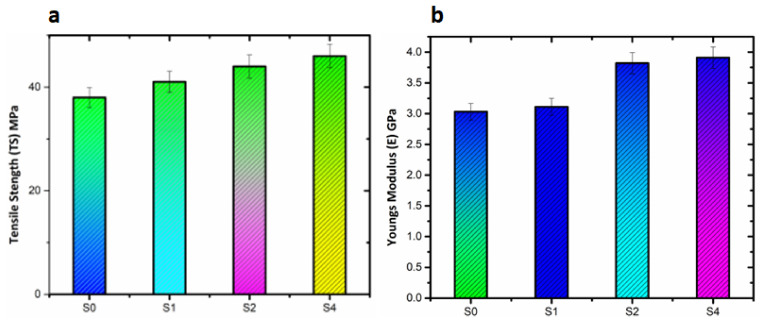
Mechanical properties of pristine PS and PS/Sepiolite composites: (**a**) Tensile Strength (TS) and (**b**) Young’s Modulus (E).

The surface morphologies of pristine PS and its nanocomposites were observed using SEM, as shown in Figure 5. Surface morphology of the neat PS is shown in Figure 5a, while Figure 5b–d present the morphologies of the 1 wt.%, 2 wt.% and 4 wt.% loadings, respectively. The cross-sectional view of PS/m-sepiolite nanocomposites in Figure 5d shows the better filler matrix cross-linking due to the VTES coupling agent, which presumably improves the dispersion of the filler in the polymer matrix composites [50]. Good interfacial adhesion and intercalation in the polymer matrix are achieved, which reflects a better dispersion of the clay particles in the polymer matrix. The clay appeared to be successfully exfoliated into the polymer matrix, depicting its increase in % crystallinity, as evident from the XRD patterns and improved mechanical properties shown in Figure 6. However, the few voids and pores at the interphase of the matrix and the filler in Figure 5b–d indicate that the clay was not completely distributed in the polymer matrix at some discrete places. This might be the result of an incomplete extrusion cycle in the twin-screw extruder or, possibly, a partial exfoliation.

### 3.4. Mechanical Properties

The mechanical properties of pristine PS and PS/m-sepiolite nanocomposites showing the yield strength (YS), tensile strength (TS), Young’s modulus (E) and elongation at break (Eb) are summarized in Table 3. The comparison among the tensile strengths of pristine PS and fabricated nanocomposites is shown in Figure 6a. The graphs indicate an increasing trend in tensile strengths of the nanocomposite. The tensile strength of pristine PS was 38.74 MPa, which increased to 46.29 MPa by adding 4 wt.% of modified sepiolite clay, indicating a better compatibility between the matrix and the filler at the interface. This can be attributed to siloxane (Si–O–C) linkages, as evident from the FTIR spectra. Similarly, the Young’s modulus (E) results of pristine PS and the nanocomposites were compared in Figure 6b, indicating the highest value with 4 wt.% loading of m-sepiolite. This increase in the mechanical properties of the nanocomposites indicates robust intermolecular interaction and better dispersion, as evident from the SEM analyses. In contrast to TS and E, Eb decreased from 2.16 to 1.61% upon addition of the m-sepiolite clay in the nanocomposites as compared to pristine PS. The decrease in Eb depicts a transition of amorphous PS toward a semicrystalline structure of the polymer nanocomposites with the addition of the filler. It was observed that the increase in filler content in the polymer matrix might have led to a rigid interphase, thereby reducing the deformability.

### 3.5. Dynamic Mechanical Analysis (DMA)

#### 3.5.1. Storage Modulus (E′)

**Figure 7:**
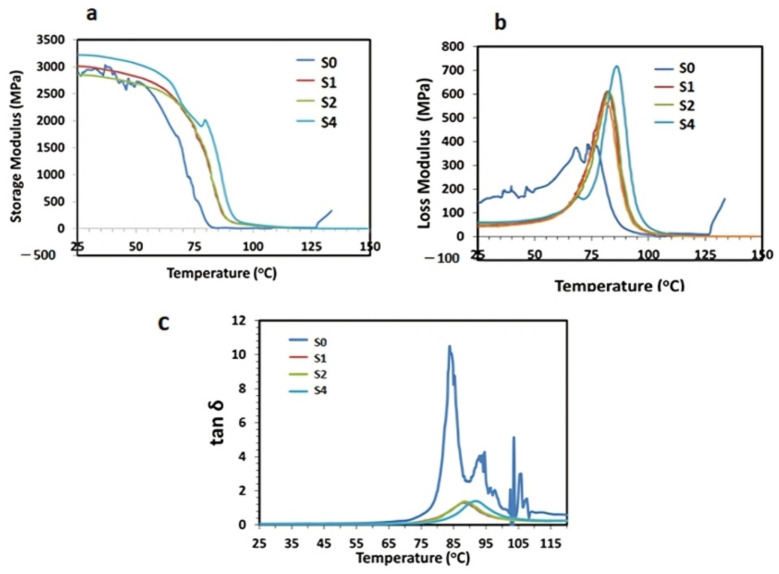
Dynamic mechanical analysis of pristine PS and PS/m-sepiolite nanocomposites: (**a**) Storage Modulus (E″); (**b**) Loss Modulus (E″); and (**c**) Damping Factor (tan δ).

The storage modulus (E′) typically indicates the material’s ability to store energy elastically. This material–property dynamic relationship gives information about the stiffness and rigidity in clay-reinforced polymer nanocomposites [51]. Figure 7a represents the three-phase transitions of pristine PS and PS/m-sepiolite nanocomposites by plotting E′ versus the temperature (25 to 150 °C) at a frequency of 1 Hz and amplitude of 15.0 μm, demonstrating the first glassy phase, second glass transition phase and third rubbery phase. The E′ of pristine PS and nanocomposites in the first phase are high due to the rigid molecular structure; however, it then decreases down to *T_g_* of pristine PS at 100 °C due to mobility in the polymeric chains at a higher temperature. Further increase in the temperature beyond *T_g_* leads to a slight variation in E′. It can be presumably attributed to the easy mobility in polymeric chains, as the viscoelastic behavior of polymer nanocomposites and interfacial adhesion due to reinforcement are affected by the movement of the polymeric chain [52].

It was noticed that at low temperature, the E′ of pristine PS was higher due to strong intermolecular interactions. The E′ increased with filler concentration, which is directly related to better compatibility at the matrix–filler interphase. The addition of m-sepiolite in the PS improved the crystallinity, thus increasing the rigidity and stiffness and resisting the mobility in polymeric chains [53]. The value of E′ decreased with an increase in temperature due to a weaker intermolecular attraction and less hindrance to molecular movement.

#### 3.5.2. Loss Modulus (E″)

The loss modulus (E″) is the amount of energy loss upon heating in response to the viscoelastic behavior of the material [54]. Figure 7b indicates the variation in the E″ of pristine PS and nanocomposites with temperature in the range of 25 to 150 °C at a constant frequency of 1 Hz. Initially, at low temperature, the E″ of pristine PS (S0) was high; however, it decreased with the loading of the filler from 1 wt.% to 4 wt.% in S1, S2 and S4 due to better interfacial adhesion at the matrix–filler interphase, which is attributed to the semi-crystalline nature inducing greater stiffness and hardness. However, as the temperature increased up to *T_g_* of PS, the value of E″ in the nanocomposites greatly increased as compared to pristine PS because of internal molecular frictions at the matrix–filler interphase, which caused a hindrance in material flow, dissipating more energy in terms of force [55]. It was further observed that the E″ decreased exponentially in the glass transition phase by observing a sharp peak as the material turned viscous in the rubbery region.

#### 3.5.3. Damping Factor (tan δ)

The damping factor (tan δ) is the ratio between the loss modulus and the storage modulus, which typically describes the material’s ability to store or dissipate energy in response to the viscoelastic behavior of polymer nanocomposites [56]. Therefore, the dependent variable tan δ versus the temperature for the pristine and polymeric nanocomposites are shown in Figure 7c. It was noticed that the tan δ peak for pristine PS was high and sharp and was significantly reduced by adding m-sepiolite clay. The results also indicate a better compatibility between the matrix and the filler due to substantial intermolecular interactions also evident from the FTIR data. Furthermore, it was observed that the value of *T_g_* was reduced for all the modified samples, which shifted the tan δ peak toward higher temperature due to mobility of the active molecules and then reduced it approaching zero. The value of *T_g_* was affected by various factors, including the intermolecular closely packed structure or the linear structure, and the nature and amount of reinforcement or filler content [57,58].

### 3.6. Thermal Gravimetric Analysis (TGA)

**Figure 8:**
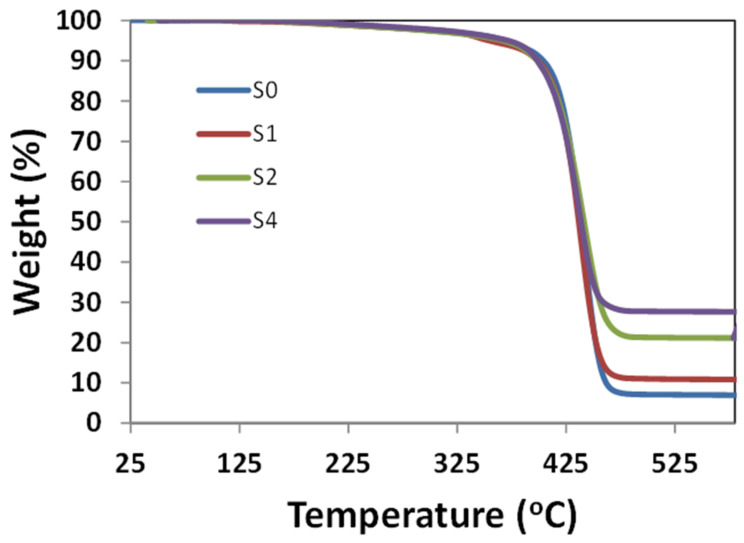
TGA analysis of pristine PS and PS/sepiolite nanocomposites.

Thermogravimetric analyses of the pristine PS and PS/sepiolite nanocomposites are shown in Figure 8. Degradation for all the samples was observed at 450 °C, as indicated by weight loss. The amounts of residue increased from 5.8 wt.% to 30.2 wt.% by incorporating m-sepiolite in S1, S2 and S4 samples, indicating an increase in thermal stability. Thus, the loading of m-sepiolite clay in the polymer matrix significantly increased the thermal stability due to a better cross-linked structure between the matrix–filler interphase, which exhibited better mechanical properties and resulted in an enhanced thermal stability.

### 3.7. Flame Retardancy Test

**Figure 9:**
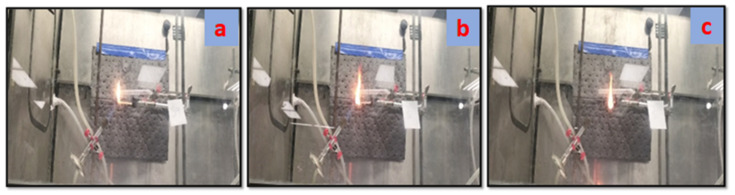
(**a**) Lighting of composite sample, (**b**) Distance of 25 mm traveled by the flame in T1, (**c**) Distance of 60 mm traveled by the flame in T2.

**Figure 10:**
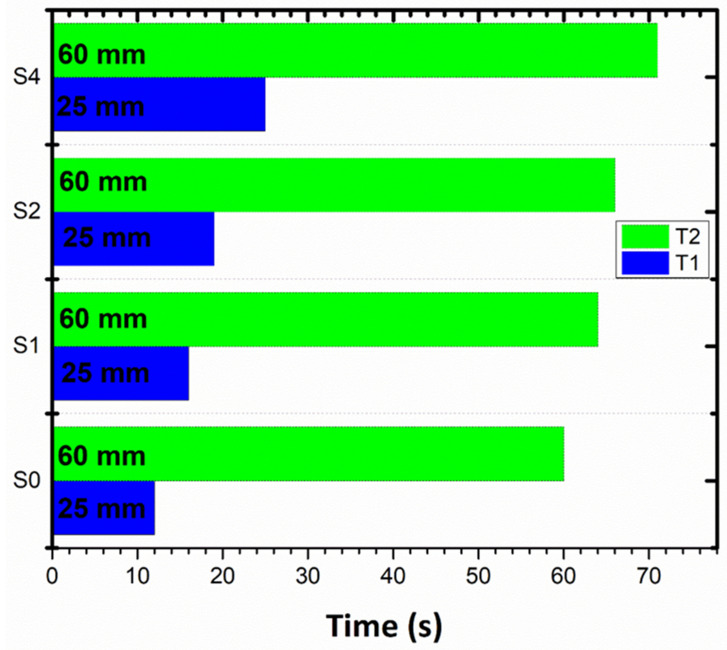
Increase in flame retardancy upon addition of m-sepiolite from S0–S4 samples in terms of T1 and T2.

The flame-retardant property of the nanocomposite samples was determined by ASTM Standard D4986-20, as shown in Figure 9. Table 4 exhibits the time taken by the flame to reach T1 and T2, marked at 25 mm and 60 mm lengths on the samples, respectively. The faster the flame travels to reach the mark (T1) and (T2), the lower the flame retardancy of the sample will be. The visuals of Figure 9a show the lighting of the composite sample with a Bunsen burner for 60 s. Figure 9b,c show the flame traveled the length of (T1) and T2, respectively, marked on the rectangular composites’ samples. The burning rate determined as per the ASTM D4986-20 standard shows a continuous decrease by incorporating the filler. Figure 10 shows a consistent increase in flame retardancy observed from pristine samples S0 to S4. A maximum decrease of 48% in the burning rate was determined for the S4 sample.

## 4. Conclusions

An inexpensive and eco-friendly material, sepiolite, was found to be a beneficial filler for developing polymeric nanocomposites using polystyrene (PS) as a matrix. FTIR and SEM analyses confirmed the successful interactions of sepiolite at the matrix–filler interphase, which resulted in good mechanical and thermal properties. The incorporation of 4 wt.% sepiolite caused an increase of 15% in the tensile strength and 25% in the Young’s modulus of polystyrene nanocomposites. A concentration higher than 4 wt.% caused an agglomeration in the nanocomposite, which can result in the loss of mechanical properties. The amounts of residue increased from 5.8 wt.% to 30.2 wt.%, reflecting the thermal stability achieved as per the TGA data. The burning rate of the fabricated sample (S4) was found to be decreased by 48%, and thus, the flame retardancy was enhanced.

## Figures and Tables

**Table 1:** Sample codes and designation of pristine polymer and polymer nanocomposite.

Sample Code	Description
**S0**	Neat polystyrene
**S1**	1 wt. % loading of m-sepiolite in polystyrene
**S2**	2 wt. % loading of m-sepiolite in polystyrene
**S4**	4 wt. % loading of m-sepiolite in polystyrene

**Table 2:** Formulation details of nanocomposites.

Sample Code	(GPPS-550 P)	M-Sepiolite	DMC	Irganox 1010	Stearic Acid
**S0**	10 g	0.0 g	0.5 g	0.1 g	0.1g
**S1**	9.9 g	0.1 g	0.5 g	0.1 g	0.1g
**S2**	9.8 g	0.2 g	0.5 g	0.1 g	0.1g
**S4**	9.6 g	0.4 g	0.5 g	0.1 g	0.1g

**Table 3:** Mechanical properties of pristine PS and PS/m-sepiolite nanocomposites.

Sample Formulation	Tensile Strength (TS) Mpa	Young’s Modulus (E) Gpa	Elongation at Break (E_b_) %
**S0**	38.74 ± 0.3	3.03 ± 0.02	2.16 ± 0.06
**S1**	41.69 ± 0.4	3.11 ± 0.06	1.99 ± 0.07
**S2**	44.91 ± 0.6	3.82 ± 0.24	1.79 ± 0.06
**S4**	46.29 ± 0.5	3.91 ± 0.05	1.61 ± 0.07

**Table 4:** Flame retardancy test data.

Sample Code	Sepiolite Loading	Time (T_1_) in Sec for (25 mm Length)	Time (T_2_) in Sec for (60 mm Length)	Burning Rate (cm/min)
**S0**	0%	12	60	50
**S1**	1%	16	64	36
**S2**	2%	19	66	31
**S4**	4%	25	71	24

## Data Availability

All the data will be available to the readers.

## References

[1] Shamsuri A.A., Jamil S.N.A. (2020). A Short Review on the Effect of Surfactants on the Mechanico-Thermal Properties of Polymer Nanocomposites. Appl. Sci..

[2] Huang G., Chen W., Wu T., Guo H., Fu C., Xue Y., Wang K., Song P. (2021). Multifunctional graphene-based nano-additives toward high-performance polymer nanocomposites with enhanced mechanical, thermal, flame retardancy and smoke suppressive properties. Chem. Eng. J..

[3] Rizal S., Fizree H., Hossain S., Gopakumar D.A., Ni E.C.W., Khalil H.A. (2020). The role of silica-containing agro-industrial waste as reinforcement on physicochemical and thermal properties of polymer composites. Heliyon.

[4] Kumar A., Sharma K., Dixit A.R. (2020). A review on the mechanical properties of polymer composites reinforced by carbon nanotubes and graphene. Carbon Lett..

[5] Hazarika D., Karak N. (2021). Fundamentals of polymeric nanostructured materials. Advances in Polymeric Nanomaterials for Biomedical Applications.

[6] Ielo I., Giacobello F., Sfameni S., Rando G., Galletta M., Trovato V., Rosace G., Plutino M. (2021). Nanostructured Surface Finishing and Coatings: Functional Properties and Applications. Materials.

[7] Ilyas R., Sapuan S., Asyraf M., Dayana D., Amelia J., Rani M., Norrrahim M., Nurazzi N., Aisyah H., Sharma S. (2021). Polymer Composites Filled with Metal Derivatives: A Review of Flame Retardants. Polymers.

[8] Karkhanis S.S., Stark N.M., Sabo R.C., Matuana L.M. (2018). Water vapor and oxygen barrier properties of extrusion-blown poly (lactic acid)/cellulose nanocrystals nanocomposite films. Compos. Part A Appl. Sci. Manuf..

[9] Jamróz E., Kulawik P., Kopel P. (2019). The Effect of Nanofillers on the Functional Properties of Biopolymer-based Films: A Review. Polymers.

[10] Soni S.K., Thomas B., Kar V.R. (2020). A Comprehensive Review on CNTs and CNT-Reinforced Composites: Syntheses, Characteristics and Applications. Mater. Today Commun..

[11] Fumagalli M., Berriot J., de Gaudemaris B., Veyland A., Putaux J.-L., Molina-Boisseau S., Heux L. (2018). Rubber materials from elastomers and nanocellulose powders: Filler dispersion and mechanical reinforcement. Soft Matter.

[12] Joo H., Jung D., Sunwoo S.-H., Koo J.H., Kim D.-H. (2020). Material Design and Fabrication Strategies for Stretchable Metallic Nanocomposites. Small.

[13] Tang M.C., Agarwal S., Alsewailem F.D., Choi H.J., Gupta R.K. (2018). A model for water vapor permeability reduction in poly (lactic acid) and nanoclay nanocomposites. J. Appl. Polym. Sci..

[14] Lee E.C., Mielewski D.F., Baird R.J. (2004). Baird, Exfoliation and dispersion enhancement in polypropylene nanocomposites by in-situ melt phase ultrasonication. Polym. Eng. Sci..

[15] Hamid Y., Tang L., Hussain B., Usman M., Liu L., Ulhassan Z., He Z., Yang X. (2021). Sepiolite clay: A review of its applications to immobilize toxic metals in contaminated soils and its implications in soil–plant system. Environ. Technol. Innov..

[16] Ladavos A., Giannakas A.E., Xidas P., Giliopoulos D.J., Baikousi M., Gournis D., Karakassides M.A., Triantafyllidis K.S. (2021). Preparation and Characterization of Polystyrene Hybrid Composites Reinforced with 2D and 3D Inorganic Fillers. Micro.

[17] Nikolaidis A.K., Achilias D.S. (2018). Thermal Degradation Kinetics and Viscoelastic Behavior of Poly (Methyl Methacrylate)/Organomodified Montmorillonite Nanocomposites Prepared via In Situ Bulk Radical Polymerization. Polymers.

[18] Ravichandran K., Praseetha P.K., Arun T., Gobalakrishnan S. (2018). Synthesis of Nanocomposites. Synthesis of Inorganic Nanomaterials.

[19] Zhu T.T., Zhou C.H., Kabwe F.B., Wu Q.Q., Li C.S., Zhang J.R. (2019). Exfoliation of montmorillonite and related properties of clay/polymer nanocomposites. Appl. Clay Sci..

[20] Wagner M.H., Narimissa E., Huang Q. (2021). Narimissa, and Q. Huang, Scaling relations for brittle fracture of entangled polystyrene melts and solutions in elongational flow. J. Rheol..

[21] Niknejad A.S., Bazgir S., Kargari A. (2021). Mechanically improved superhydrophobic nanofibrous polystyrene/high-impact polystyrene membranes for promising membrane distillation application. J. Appl. Polym. Sci..

[22] Hirayama D., Nunnenkamp L.A., Braga F.H.G., Saron C. (2021). Enhanced mechanical properties of recycled blends acrylonitrile–butadiene–styrene/high–impact polystyrene from waste electrical and electronic equipment using compatibilizers and virgin polymers. J. Appl. Polym. Sci..

[23] Bhattacharya M. (2016). Polymer Nanocomposites-A Comparison between Carbon Nanotubes, Graphene, and Clay as Nanofillers. Materials.

[24] Jlassi K., Chandran S., Mičušik M., Benna-Zayani M., Yagci Y., Thomas S., Chehimi M.M. (2015). Poly (glycidyl methacrylate)-grafted clay nanofiller for highly transparent and mechanically robust epoxy composites. Eur. Polym. J..

[25] Paszkiewicz S., Pypeć K., Irska I., Piesowicz E. (2020). Functional Polymer Hybrid Nanocomposites Based on Polyolefins: A Review. Processes.

[26] Majka T.M., Pielichowski K. (2019). Functionalized Clay-Containing Composites. Polymer Composites with Functionalized Nanoparticles.

[27] Niroumand H., Zain M., Alhosseini S.N. (2013). The Influence of Nano-clays on Compressive Strength of Earth Bricks as Sustainable Materials. Procedia-Soc. Behav. Sci..

[28] Chiu C.-W., Huang T.-K., Wang Y.-C., Alamani B.G., Lin J.-J. (2014). Intercalation strategies in clay/polymer hybrids. Prog. Polym. Sci..

[29] LeBaron P. (1999). Polymer-layered silicate nanocomposites: An overview. Appl. Clay Sci..

[30] Wang K.H., Choi M.H., Koo C.M., Choi Y.S., Chung I.J. (2001). Synthesis and characterization of maleated polyethylene/clay nanocomposites. Polymer.

[31] Galan E. (1996). Properties and applications of Fpalygroskite-sepiolite clays. Clay Miner..

[32] Hibino T., Tsunashima A., Yamazaki A., Otsuka R. (1995). Model Calculation of Sepiolite Surface Areas. Clays Clay Miner..

[33] Preisinger A. (1961). Sepiolite and Related Compounds: Its Stability and Application. Clays Clay Miner..

[34] Fajdek-Bieda A., Wróblewska A., Miądlicki P., Szymańska A., Dzięcioł M., Booth A.M., Michalkiewicz B. (2019). Influence of Technological Parameters on the Isomerization of Geraniol Using Sepiolite. Catal. Lett..

[35] Bilotti E., Zhang R., Deng H., Quero F., Fischer H.R., Peijs T. (2009). Sepiolite needle-like clay for PA6 nanocomposites: An alternative to layered silicates?. Compos. Sci. Technol..

[36] Zaini N.A.M., Ismail H., Rusli A. (2017). Short Review on Sepiolite-Filled Polymer Nanocomposites. Polym. -Plast. Technol. Eng..

[37] Tahira F., Ali M., Shafiq M., Yasin T. (2016). Synthesis and Characterization of Nanocomposites Based on Styrene Butadiene Rubber/Sepiolite. Asian J. Chem..

[38] Ongen A., Ozcan H.K., Özbaş E.E., Balkaya N. (2012). Adsorption of Astrazon Blue FGRL onto sepiolite from aqueous solutions. Desalination Water Treat..

[39] Altaf F., Batool R., Ahmad M.A., Raza R., Khan M.A., Abbas G. (2018). Novel vinyl-modified sepiolite-based polymer nanocomposites: Synthesis and characterization. Iran. Polym. J..

[40] Chen H., Zheng M., Sun H., Jia Q. (2007). Characterization and properties of sepiolite/polyurethane nanocomposites. Mater. Sci. Eng. A.

[41] Shafiq M., Yasin T., Saeed S. (2012). Synthesis, and characterization of linear low-density polyethylene/sepiolite nanocomposites. J. Appl. Polym. Sci..

[42] Ekici S., Işıkver Y., Saraydın D. (2006). Poly (Acrylamide-Sepiolite) Composite Hydrogels: Preparation, Swelling and Dye Adsorption Properties. Polym. Bull..

[43] Sabbar A.N., Mohammed H.S., Ibrahim A.R., Saud H.R. (2019). Thermal and Optical Properties of Polystyrene Nanocomposites Reinforced with Soot. Orient. J. Chem..

[44] Fang J., Xuan Y., Li Q. (2010). Preparation of polystyrene spheres in different particle sizes and assembly of the PS colloidal crystals. Sci. China Technol. Sci..

[45] Menta V.G.K., Tahir I., Abutunis A. (2022). Effects of Blending Tobacco Lignin with HDPE on Thermal and Mechanical Properties. Materials.

[46] Gupta P., Bera M., Maji P.K. (2017). Nanotailoring of sepiolite clay with poly [styrene-b-(ethylene-co-butylene)-b-styrene]: Structure-property correlation. Polym. Adv. Technol..

[47] Ding P., Qu B. (2006). Synthesis, and characterization of polystyrene/layered double-hydroxide nanocomposites viain situ emulsion and suspension polymerization. J. Appl. Polym. Sci..

[48] Suresh K., Kumar R.V., Pugazhenthi G. (2016). Processing and characterization of polystyrene nanocomposites based on CoAl layered double hydroxide. J. Sci. Adv. Mater. Devices.

[49] Panwar A., Choudhary V., Sharma D. (2013). Role of compatibilizer and processing method on the mechanical, thermal and barrier properties of polystyrene/organoclay nanocomposites. J. Reinf. Plast. Compos..

[50] Mir S., Yasin T., Halley P.J., Siddiqi H.M., Ozdemir O., Nguyen A. (2013). Thermal, and rheological effects of sepiolite in linear low-density polyethylene/starch blend. J. Appl. Polym. Sci..

[51] Jlassi K., Krupa I., Chehimi M.M., Jlassi K., Chehimi M.M., Thomas S. (2017). Chapter 1-Overview: Clay Preparation, Properties, Modification. Clay-Polymer Nanocomposites.

[52] He L., Xia F., Wang Y., Yuan J., Chen D., Zheng J. (2021). Mechanical and Dynamic Mechanical Properties of the Amino Silicone Oil Emulsion Modified Ramie Fiber Reinforced Composites. Polymers.

[53] Mittal M., Chaudhary R. (2018). Effect of fiber content on thermal behavior and viscoelastic properties of PALF/Epoxy and COIR/Epoxy composites. Mater. Res. Express.

[54] Mercy J.L., Prakash S. (2019). Investigation of damage processes of a microencapsulated self-healing mechanism in glass fiber-reinforced polymers. Modelling of Damage Processes in Bio composites, Fiber-Reinforced Composites and Hybrid Composites.

[55] Bindu P., Thomas S. (2013). Viscoelastic behavior, and reinforcement mechanism in rubber nanocomposites in the vicinity of spherical nanoparticles. J. Phys. Chem. B.

[56] Wei W., Zhang Y., Liu M., Zhang Y., Yin Y., Gutowski W.S., Deng P., Zheng C. (2019). Improving the Damping Properties of Nanocomposites by Monodispersed Hybrid POSS Nanoparticles: Preparation and Mechanisms. Polymers.

[57] Zhu G.-L., Han D., Yuan Y., Chen F., Fu Q. (2018). Improving Damping Properties and Thermal Stability of Epoxy/Polyurethane Grafted Copolymer by Adding Glycidyl POSS. Chin. J. Polym. Sci..

[58] Sun L., Gibson R.F., Gordaninejad F., Suhr J. (2009). Energy absorption capability of nanocomposites: A review. Compos. Sci. Technol..

